# Removal of Cd(II), Cu(II), and Pb(II) by adsorption onto natural clay: a kinetic and thermodynamic study

**DOI:** 10.3906/kim-2004-82

**Published:** 2021-04-28

**Authors:** Brahim ABBOU, Imane LEBKIRI, Hanae OUADDARI, Lamya KADIRI, Abdelkarim OUASS, Amar HABSAOUI, Ahmed LEBKIRI, El Housseine RIFI

**Affiliations:** 1 Department of Chemistry, Faculty of Science, Ibn Tofail University, Kenitra Morocco2; 2 Department of Chemistry, Faculty of Science, Hassan II University, Mohammedia Morocco

**Keywords:** Clay, heavy metals, adsorption, isotherm, FTIR, thermodynamic

## Abstract

In this work, we study the elimination of three bivalent metal ions (Cd^2+^, Cu^2+^, and Pb^2+^) by adsorption onto natural illitic clay (AM) collected from Marrakech region in Morocco. The characterization of the adsorbent was carried out by X-ray fluorescence, Fourier transform infrared spectroscopy and X-ray diffraction. The influence of physicochemical parameters on the clay adsorption capacity for ions Cd^2+^, Cu^2+^, and Pb^2+^, namely the adsorbent dose, the contact time, the initial pH imposed on the aqueous solution, the initial concentration of the metal solution and the temperature, was studied. The adsorption process is evaluated by different kinetic models such as the pseudo-first-order, pseudo-second-order, and Elovich. The adsorption mechanism was determined by the use of adsorption isotherms such as Langmuir, Freundlich, and Temkin models. Experiments have shown that heavy metals adsorption kinetics onto clay follows the same order, the pseudo-second order. The isotherms of adsorption of metal cations by AM clay are satisfactorily described by the Langmuir model and the maximum adsorption capacities obtained from the natural clay, using the Langmuir isotherm model equation, are 5.25, 13.41, and 15.90 mg/g, respectively for Cd(II), Cu(II), and Pb(II) ions. Adsorption of heavy metals on clay is a spontaneous and endothermic process characterized by a disorder of the medium. The values of ΔH are greater than 40 kJ/mol, which means that the interactions between clay and heavy metals are chemical in nature.

## 1. Introduction

Natural water resources are becoming increasingly scarce. Thus, the state of the environment has become one of humanity’s major concerns because of its degradation. This degradation is mainly due to industrial development which generates effluents discharged into the receiving environment without any treatment in most cases. These releases consist of toxic chemical elements and compounds, including heavy metals, which pose a serious threat to our environment and impair water quality. Currently, they are of great concern because of their toxicity to ecosystems and their harmful effects on human health. Cadmium, copper, and lead are considered to be dangerous micropollutants [1], the toxicity caused by these metals is considered to be high even to the state of traces [2]. Many cleaning methods and techniques have been developed in recent years to remove heavy metals from polluted waters. These techniques include chemical precipitation processes, flocculation, filtration, ion exchange, membrane processes, and adsorption [3–8]. The most used and studied method is adsorption method, due to its ease of use and the high availability and abundance of natural adsorbents [9–12]. Activated carbons are among the most widely used materials due to their high adsorption capacity [13], but they have numerous disadvantages, including high cost, intraparticle resistance in the adsorption process, low mechanical strength, and difficulty to regenerate. [14].

Natural clay minerals have recently received considerable attention as alternative materials that are less expensive, nontoxic, and abundant and that have multifunctional properties depending on the type of clay [15]. The main advantages of using these materials are due to their different characteristics, low cost, and abundant availability.

The aim of this study is the valorization of a Moroccan natural clay as an adsorbent for the removal of cadmium, copper, and lead from synthetic aqueous solutions. The adsorbent was characterized using the X-ray fluorescence (XRF), Fourier transform infrared spectroscopy (FTIR), and X-ray diffraction (XRD) methods. To better understand the nature of the reaction mechanisms involved in the adsorption phenomenon, the linear shapes of different kinetic and isothermal models were calculated and evaluated.

## 2. Materials and methods

### 2.1. Characterization techniques

AM clay sample was identified using different characterization techniques. The chemical composition for AM clay was determined with XRF using an Axios-Panalytical device. XRD analyses were carried out using a PANalytical X’Pert HighScore Plus diffractometer, using Cu-Kα radiation (1.5418 Å) at a goniometer rate of 2θ = 4°/min. The FTIR analysis of AM clay was performed using a Vertex 70 spectrometer (Rabat, Morocco). The analysis was done by scanning from 4000 cm^-1^ to 400 cm^-1^ with a resolution of 4 cm^-1^.

### 2.2. Adsorbent 

The adsorbent used in this study is a natural clay material collected from Marrakech region, southern of Morocco. The clay material, which has not undergone any physical or chemical pretreatment, was crushed and sieved to obtain lower fractions below 120 µm. The particles were then dried at 100 °C for 24 h and used for further experiments.

### 2.3. Adsorbate

The stock solutions of each metal ion (Cd^2+^, Cu^2+^, or Pb^2+^) with a concentration of 1000 mg/L are prepared separately by dissolving a specific amount of the corresponding metal salt (Cd(NO_3_)_2_, CuSO_4_ 5H_2_O, or Pb(NO_3_)_2_, purchased from Sigma–Aldrich (Darmstadt, Germany) and Solvachim (Casablanca, Morocco) in distilled water. The desired working concentrations are prepared by diluting the stock solutions.

### 2.4. Adsorption experiments

The adsorption behavior of AM clay towards metal ions Cd^2+^, Cu^2+^, and Pb^2+^ has been studied with batch method under various experimental conditions, such as adsorbent dose (0.1 to 1 g), contact time (from 0 to 180 min), pH of the solution (2 to 6), initial concentration of the adsorbate (from 0 to 200 mg/L), and temperature (25 to 55 °C). After adsorption, AM clay was separated from the liquid phase using a polytetrafluoroethylene (PTFE) membrane filter (Sartorius) with a pore size of 0.45 µm, the filtrate was recovered and analyzed by inductively coupled plasma atomic emission spectroscopy. The data obtained from the adsorption tests are used for the calculation of the adsorption efficiency R% and the adsorption capacity q (mg/g) by Eq. (1) and Eq. (2), respectively:

(1)R%=(C0-Cr)C0

(2)q=(C0-Cr)mV

where C_0_ (mg/L) is the initial concentration of adsorbate, C_r_ (mg/L) is the residual concentration of adsorbate, V (L) is the volume of the solution, and m (g) is the amount of adsorbent.

## 3. Results and discussion

### 3.1. Characterization of the adsorbent

The elemental chemical analysis of the natural clay presented in Table 1 shows that silica and alumina are the predominant constituents. They are found in a SiO_2_/Al_2_O_3_ ratio equal to 3.64, the relatively high ratio is an indication of the presence of free quartz in the clay fraction in large proportion [16]. Fe_2_O_3_, MgO, K_2_O, and Na_2_O are present in small quantities in the sample. Other oxides are present in the sample as impurities such as TiO_2_, P_2_O_5_, and SO_3_. The low CaO content indicates a low amount of calcium carbonate [17]. The loss on ignition (LoI) at 1000 °C was 5.77% by mass. It is due to the decomposition of carbonates and dehydroxylation of clay minerals [18].

**Table 1:** Chemical composition of AM clay.

Elemental composition	Weight %	Elemental composition	Weight %
SiO2	65.80	Na2O	1.07
Al2O3	18.10	CaO	0.78
Fe2O3	3.56	TiO2	0.76
K2O	2.12	P2O5	0.17
MgO	1.41	SO3	0.12
LoI*	5.77		

*Loss on ignition

**Figure 1:**
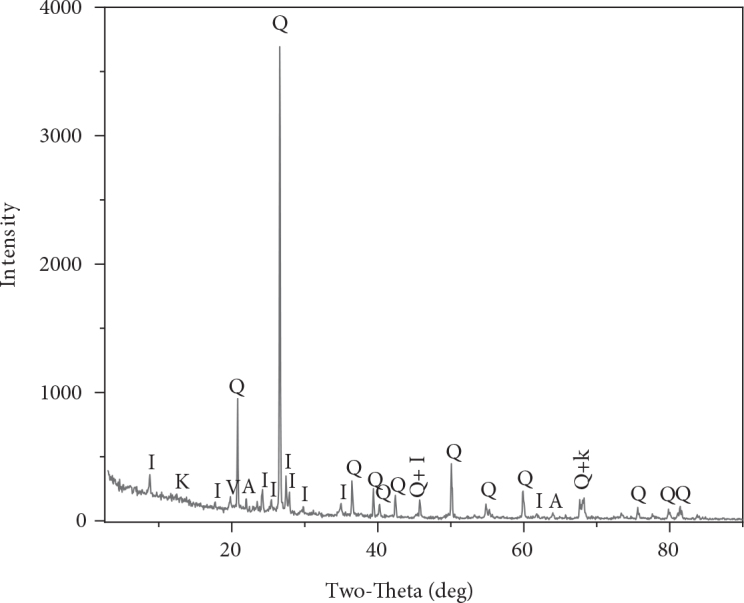
X-ray diffraction pattern of AM clay. (A = albite; I = illite; K= kaolinite; V = vermiculite; Q = quartz).

The mineralogical composition of AM clay was determined from the X-ray diffractogram represented in Figure 1. The XRD patterns indicate that our sample is essentially composed of illite as principal phase and a considerable presence of quartz in nonnegligible quantity with the existence of a small amount of other associated phases such as: kaolinite, albite and vermiculite.

**Figure 2:**
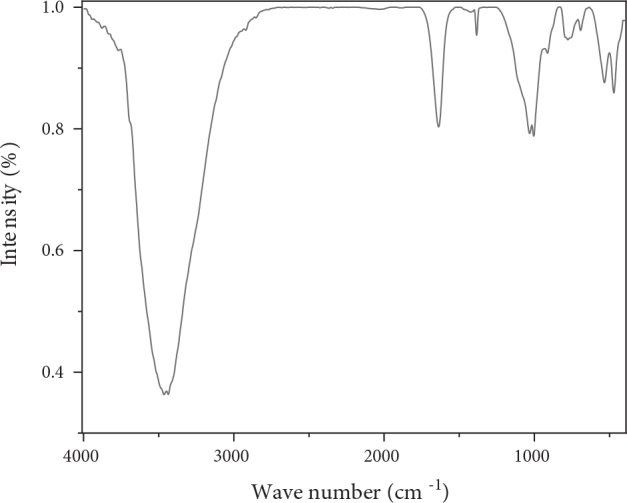
Infrared spectrum of AM clay.

The FTIR presented in Figure 2 shows a band that ranges between 3200 and 3800 cm^-1^ located at 3436 cm^-1^ corresponding to the stretching vibrations of the internal OH groups of water molecule [9,16] , the wide band at 1637 cm^-1^ and the band at 1381 cm^-1^ are attributed to the deformation of H_2_O [19,20]. The bands located at 693, 776, and 1005 cm^-1^ and the intense band located between 900 and 1200 cm^-1^ and centered at 1031 cm^-1^ correspond to the stretching vibrations of Si-O [9,20–24]. Intense peaks were observed around 472 and 533 cm^-1^ attributable to the deformation of Si–O–Mg and Si–O–Al, respectively [25,26]. The band located at 912 cm^-1^ is attributed to the bending vibrations of the groups Al–Al–OH and Al–Mg–OH [27,28]. The organic matter content is practically nil given the absence of the IR bands relating to the aliphatic and aromatic groups.

### 3.2. Adsorption experiments

#### 3.2.1. Effect of adsorbent dose

**Figure 3:**
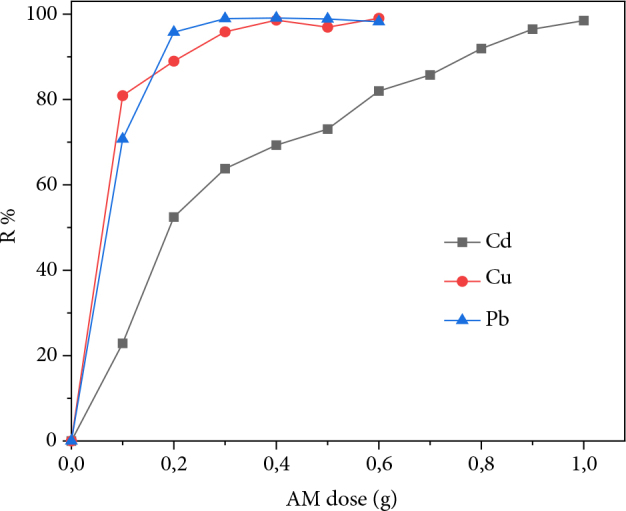
Evolution of heavy metal removal efficiency as a function of AM clay dose.

In order to determine the optimal masses to be used for the adsorption tests, we studied the clay dose effect on the adsorption efficiency. For this purpose, masses of AM clay (0.1 to 1g) are each brought into contact with a metal solution containing either Cd^2+^, Cu^2+^, or Pb^2+^ ions. Figure 3 shows the evolution of the adsorption efficiency of the three metals as a function of AM clay mass.

It can be seen that the adsorption efficiency of the uptake of metal for the three metal solutions increases progressively as the mass of AM clay increases. This is due to the increase in specific surface area and the adsorption sites attributed to the increase in the adsorbent mass [29]. For copper and lead, we note that 0.2 g of AM clay is sufficient to recover 100% of each metal. On the other hand, total cadmium removal requires four times as much support. 

#### 3.2.2. Contact time effect

**Figure 4:**
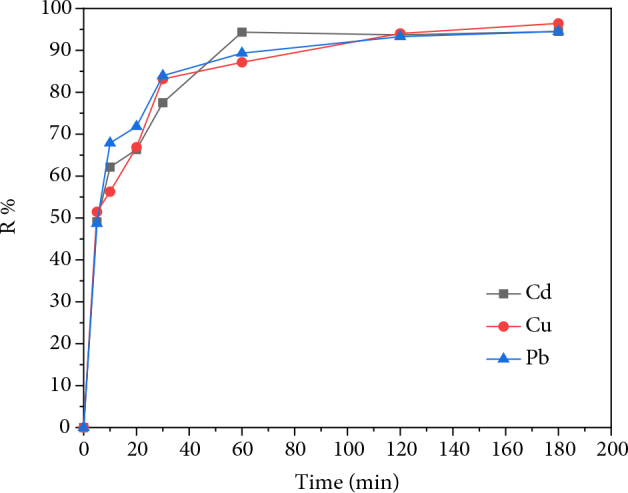
Effect of time on the adsorption efficiency of heavy metals onto AM clay.

Contact time is an important parameter that controls the effectiveness of the adsorption phenomenon as shown in Figure 4, which represents the evolution of the adsorption efficiency as a function of contact time.

The amount of metal adsorbed by AM clay of the three metal solutions indicates the presence of a high affinity with AM clay from the first minutes of contact of the two phases. It can be seen that more than 75% of the initial charge of each metal is adsorbed after 40 min, followed by a slow increase until equilibrium is reached. This can be interpreted by the fact that at the beginning of adsorption, the number of active sites available on the surface of AM clay is much greater than the number of sites remaining after a certain contact time [30]. The equilibrium times of the adsorption of the three metals are as follows: 60 min for cadmium and 120 min for copper and lead. The removal rate of the three metals is around 94%. 

#### 3.2.3. Effect of solution pH on adsorption

**Figure 5:**
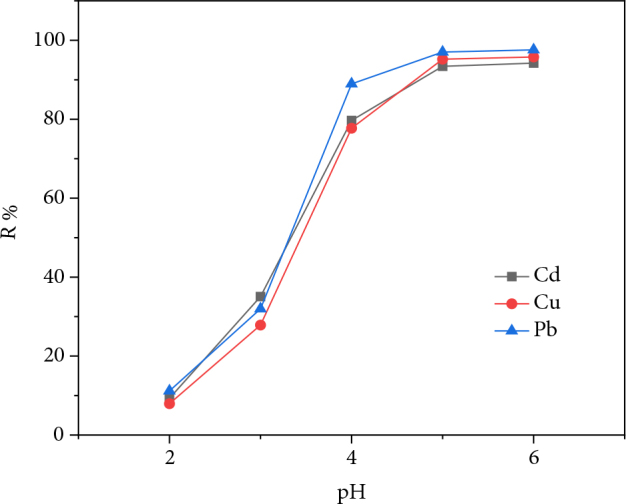
Variation of removal efficiency of heavy metals as a function of the initial pH of the solution.

The adsorption of metal ions is a phenomenon that is strongly influenced by pH. This is due to the involvement of mechanisms that are dependent on pH such as ion exchange or retention by electrostatic forces. The adsorption of the three metal ions on AM clay at different pH is shown in Figure 5. 

We notice that the adsorption efficiency of the material increases with increasing pH. Thus, at acidic pH (pH = 2), the adsorption efficiency is too low; 9.31%, 7.93%, and 11.16% for cadmium, copper, and lead, respectively. The low adsorption efficiency of AM clay at acidic pH can be explained by the lack of electrostatic attraction to trap metal cations because of the positive charges it carries at this pH. In addition, the competitive effect of H+ present in the acid solution: hydronium ions are more adsorbed than metal ions due to their high mobility. At slightly acidic pH (from 4 to 6), adsorption is more pronounced and the adsorption efficiency increases with increasing pH, at pH = 5, the following values are recorded: 93.42%, 95.21%, and 97.02% for cadmium, copper, and lead, respectively. The mechanism involved at this pH range is an ion exchange that occurs between the metal cations and the Na^+^, K^+^, Ca^2+^, Mg^2+^ cations located in the AM clay exchange sites [31]. The almost total elimination of the metal cations Cd^2+^, Cu^2+^, and Pb^2+^ is obtained beyond pH = 5.

#### 3.2.4. Initial concentration effect

**Figure 6:**
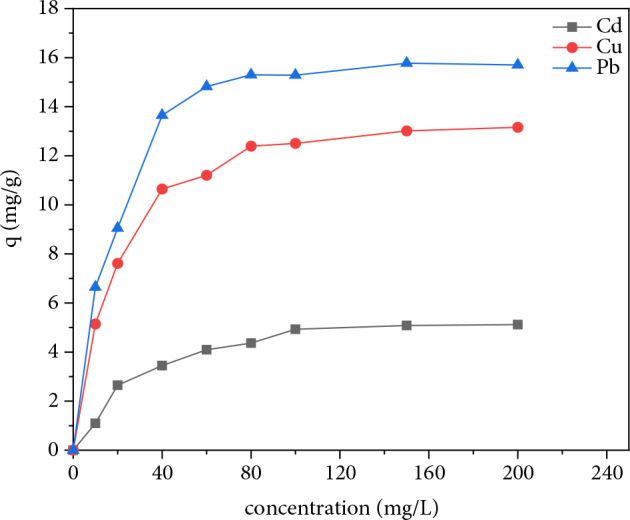
Variation in the adsorption capacity of heavy metals as a function of initial concentration.

The initial concentration provides an important driving force to overcome all the mass transfer resistances of all molecules between the aqueous and solid phases [32–34]. The study of initial concentration effect makes it possible to deduce the effectiveness of the present adsorption system with metal solutions of varying concentrations. In addition, it will allow the study of the mechanism involved through different adsorption isotherms. In this study, the effect of the initial concentration of metal solutions on the amount adsorbed (mg/g) per AM clay was investigated over a range of initial concentrations from 0 to 200 mg/L. The adsorption capabilities of metal solutions are shown in Figure 6.

 Monitoring the initial charge effect shows that the adsorption capacity at equilibrium increases with the increase of the initial metal charge, this increase is over when the clay support reaches its maximum adsorption capacity and becomes saturated with the adsorbed metal. In fact, at low initial concentrations, the adsorption sites at the clay support are vacant and tend to fix more metal ions. In general, the amount of metal adsorbed increases with increasing initial concentrations of the metal solution and then decreases to reach a plateau corresponding to saturation of the adsorption sites. The threshold characterizing the maximum adsorption capacity is generally reached from the initial concentration of 100 mg/L for cadmium, 80 mg/L for copper, and 60 mg/L for lead. The maximum adsorption capacities values obtained for cadmium, copper, and lead are 5.12, 13.16, and 15.70 mg/g, respectively.

#### 3.2.5. Temperature effect

**Figure 7:**
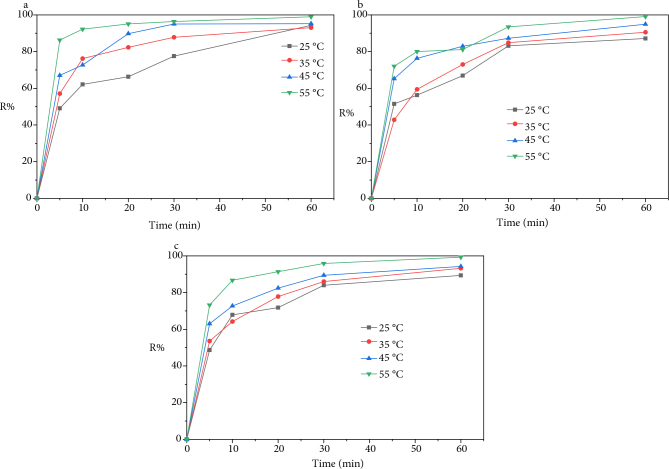
Effect of temperature on the absorption of metal ions.

Adsorption is a process that can be exothermic or endothermic. Therefore, we monitored the impact of temperature on the adsorption of the three metals onto AM clay for the following temperatures: 25, 35, 45, and 55 °C. Figure 7 shows the variations in the adsorption efficiency of Cd^2+^, Cu^2+^, and Pb^2+^. 

According to these curves it can be seen that temperature has a positive effect on adsorption. An increase in temperature improves the adsorption capacity of metal ions by AM clay, indicating an endothermic nature of adsorption. The increase in the adsorption capacity of the clay support with increasing temperature can be attributed either to the increase in the number of active sites available on the surface of the support, or to the increase in the mobility of metal cations in the solution [11].

#### 3.2.6. Selectivity

**Figure 8:**
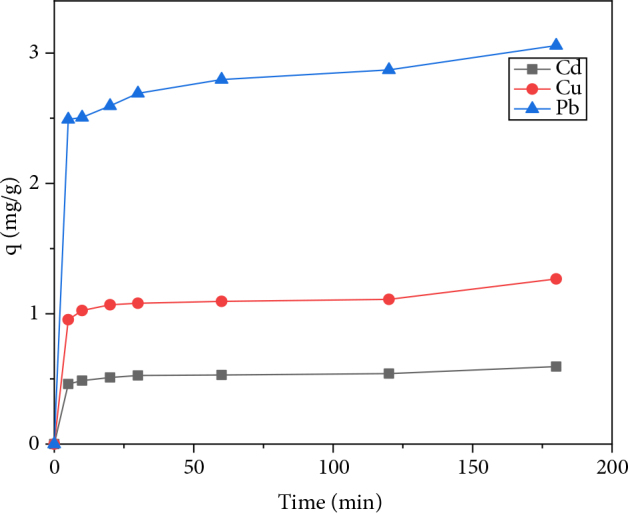
Effect of time on the competitive adsorption of heavy metals on AM clay.

To determine the selectivity order of the three heavy metals on AM clay competitive adsorption experiment was conducted. The experiment was carried out by stirring 0.5 g of AM clay in a 100-mL solution containing 10–4 M of each metal ion. Figure 8 shows the variations in adsorption capacities for the three metal cations as a function of time. The curves follow the same pace; there is an increase in adsorption capacity over time until reaching the equilibrium where the curve tends towards a time independent level. 

The adsorption capacities for the three metals are respectively: 0.59, 1.27, and 3.06 mg/g for cadmium, copper, and lead. The results show that the metal ion Pb^2+^ has a high affinity to AM clay adsorption compared to other metal ions (Cd^2+^ and Cu^2+^). The adsorption selectivity of these three bivalent metals by the AM clay follows the following order: Pb^2+^ > Cu^2+^ > Cd^2+^ [12]. The same selectivity order was obtained by Li et al. for the adsorption of Pb(II), Cu(II), and Cd(II) onto White pottery clay [35].

The selectivity order of some heavy metals on different natural clay along with AM clay is reported in Table 2. As is seen in Table 2, the selectivity order depends on the type and properties of clay.

**Table 2:** Selectivity order of some heavy metals on various natural clay.

Clay	Selectivity order	References
Ca-montmorillonite	Cr3+ > Cu^2+^ > Zn2+ > Cd^2+^ > Pb^2+^	[36]
Illite	Cr3+ > Pb^2+^ > Cu^2+^ > Zn2+ > Cd^2+^	[36]
Kaolinte	Pb^2+^ > Cd^2+^ > Ni2+ > Cu^2+^	[37]
Kaolin	Cr3+ > Zn2+ > Cu^2+^ ≈ Cd^2+^ ≈ Ni2+ > Pb^2+^	[38]
Ball clay	Cd^2+^ > Cu^2+^ > Ni2+ > Zn2+ > Pb^2+^ > Cr3+	[12]
White pottery clay	Pb^2+^> Cu^2+^ > Cd^2+^	[35]
AM clay	Pb^2+^> Cu^2+^ > Cd^2+^	Present study

### 3.3. Adsorption kinetics

For the kinetic study of the adsorption process, three kinetic models namely, the pseudo-first-order, the pseudo-second-order, and Elovich, are selected in this study to describe the process of adsorption. The pseudo-first-order equation is given by Eq. (3) [39] :

(3)log(qe-qt)=logqe-k12.303t

the pseudo-second-order kinetic model is expressed by Eq. (4) [40] :

(4)tqt=1k2qe2+1qet

where q_e_ (mg/g) and qt (mg/g) are respectively the amounts of M^2+^ adsorbed on AM clay at equilibrium and at time t (min). k_1_ (min^-1^) and k_2_ (g/mg min^-1^) are the pseudo-first-order and pseudo-second-order rate constants, respectively. 

Elovich kinetic model is one of the most widely used models to verify and describe chemisorption adsorption. it is expressed by the following equation (Eq. 5) [41]:

(5)qt=1βln(αβ)+1βlnt

where α (mg g^-1^ min^-1^) is the initial adsorption rate and β (g mg^-1^) is the desorption constant related to the extent of surface coverage and activation energy for chemisorption.

The results of the adjustment of these three models are presented in Table 3. For both models, pseudo-first-order and Elovich, the experimental data deviated significantly from linearity, as confirmed by the low values of correlation coefficients R^2^_1_ and R^2^_E_ and the values of the calculated capacities (q_e,cal_) which are smaller than the experimental q_e_ values. Therefore, the models of the pseudo-first-order and Elovich are inapplicable to this system. In contrast, the values of q_e,cal_ determined from the pseudo-second-order kinetic model are in good agreement with the experimental results and the correlation coefficients R_2_^2^ are close to unity. The applicability of the pseudo-second-order kinetic model suggests that the adsorption of M^2+^ on AM clay is based on a chemical reaction (chemisorption), involving an exchange of electrons between the adsorbent and the adsorbate [42,43].

**Table 3:** Kinetic parameters for Cd(II,) Cu(II), and Pb(II).

Concentration (mg/L)			10	20	40	60	80	100	150	200
[Cd^2+^]	qe (exp)		1.10	-	3.45	4.09	4.37	4.93	4.92	5.12
	pseudo-first-order	qe (cal)	0.75	-	1.63	0.78	0.85	1.53	2.01	1.88
		K1	0.0539	-	0.0239	0.0387	0.0366	0.0190	0.0211	0.0275
		R12	0.9444	-	0.7992	0.6750	0.7933	0.6284	0.7619	0.8306
	pseudo-second-order	qe (cal)	1.14	-	3.54	4.14	4.39	4.96	5.00	5.19
		K2	0.1371	-	0.0456	0.1301	0.1981	0.0578	0.0394	0.0501
		R22	0.9989	-	0.9995	0.9995	0.9999	0.9991	0.9992	0.9989
	Elovich	α	1.3862	-	4.1336	157.4839	2.83E+10	2017.6545	30.1701	1.43E+04
		β	6.3572	-	2.0121	2.5336	7.0824	2.7966	1.8206	3.1313
		RE2	0.9352	-	0.8878	0.4992	0.9576	0.9016	0.8643	0.9530
[Cu^2+^]	qe (exp)		5.15	-	10.73	11.21	12.39	12.51	13.01	13.16
	pseudo-first-order	qe (cal)	3.00	-	6.05	4.63	5.32	8.56	10.34	8.30
		K1	0.0539	-	0.0367	0.0238	0.0181	0.0143	0.0299	0.0102
		R12	0.9444	-	0.9669	0.8068	0.6751	0.9014	0.9446	0.7683
	pseudo-second-order	qe (cal)	4.57	-	11.09	11.40	12.55	13.12	13.93	13.25
		K2	0.0343	-	0.0156	0.0185	0.0133	0.0044	0.0051	0.0046
		R22	0.9989	-	0.9993	0.9995	0.9980	0.9867	0.9915	0.9712
	Elovich	α	5.5447	-	53.4014	105.8550	33.5625	3.1631	6.6716	4.9754
		β	1.5893	-	0.8143	0.8430	0.6560	0.4739	0.5027	0.5165
		RE2	0.9352	-	0.9644	0.9387	0.9008	0.9619	0.9199	0.9273
[Pb^2+^]	qe (exp)		-	9.05	14.08	15.45	16.23	15.29	15.77	15.84
	pseudo-first-order	qe (cal)	-	4.53	9.69	8.11	9.38	9.24	6.72	12.66
		K1	-	0.0327	0.0343	0.0313	0.0300	0.0200	0.0324	0.0417
		R12	-	0.9217	0.8869	0.8390	0.9064	0.8783	0.8209	0.9226
	pseudo-second-order	qe (cal)	-	9.31	15.11	16.35	16.95	15.96	16.18	17.20
		K2	-	0.0203	0.0057	0.0067	0.0075	0.0057	0.0135	0.0045
		R22	-	0.9998	0.9987	0.9948	0.9995	0.9977	0.9997	0.9968
	Elovich	α	-	21.6325	3.1958	5.5083	9.0472	6.1802	29.1158	3.3486
		β	-	0.8512	0.3456	0.3389	0.3699	0.3884	0.4587	0.3058
		RE2	-	0.9152	0.9391	0.7953	0.9165	0.9360	0.8365	0.9358

### 3.4. Adsorption isotherms

In order to understand precisely the mechanisms involved during the adsorption of metal ions (Cd^2+^, Cu^2+^, and Pb^2+^) on the clay support, we sought to model the adsorption isotherms by applying the most commonly used models: Langmuir, Freundlich, and Temkin. The Langmuir model is based on the assumption that the surface is uniform with no interactions between the adsorbed molecules and that it has a defined adsorption sites. [44] . 

Langmuir linear form is given by the following equation (Eq. 6):

(6)Ceqe=1KLqm+Ceqm

where q_m_ is maximum adsorbed capacity (mg/g), K_L_ is equilibrium constant characteristic of the adsorbent (L/mg) dependent on temperature and experimental conditions, and C_e_ is equilibrium adsorbate concentration (mg/L). 

By plotting C_e_/q_e_ as a function of C_e_, we obtain a slope line 1/qm and ordinate at the origin 1/K_L_.q_m_. 

The separation factors constant RL used to ascertain the Langmuir model, which is defined by the following equation (Eq. 7) [45]:

(7)RL=1(1+KLC0)

where C_0_ is the initial concentration (mg/L) and K_L_ is the Langmuir constant (L/mg). 

The values obtained are interpreted as follows: 

- R_L_ > 1 indicates that the adsorption is unfavorable.

- R_L_ = 1 indicates that the adsorption is linear.

- 0 < R_L_ < 1 indicates that the adsorption is favorable.

- R_L_ = 0 indicates that the adsorption is irreversible.

The Freundlich model assumes that the adsorbent surface is heterogeneous with a nonuniform energy distribution of adsorption sites on the surface. This model admits the existence of interactions between the adsorbed molecules [46]. Freundlich linear equation is given by the following equation (Eq. 8):

(8)logqe=logKF+1nlogCe

where K_F_ is the adsorption capacity, 1/n is the adsorption intensity, q_e_ is the amount of solute adsorbed per unit mass of adsorbent at equilibrium (mg/g), and C_e_ is the concentration of the solute in the solution at equilibrium (mg/L). 

By plotting log(q_e_) as a function of log(C_e_), we obtain a straight line with slope 1/n and ordinate at origin logK_F_.

The Temkin isotherm is based on the assumption that the free energy of sorption is a function of the surface coverage. The linear form is written as in Eq. (9) [47,48]:

(9)qe=BlnKT+BlnCe

where B = (RT)/b_T_, where T is the absolute temperature (K) and R is the universal gas constant (8,314 J.mol^-1^ K ^-1^), the constant bT (J/mol) is the heat of adsorption and KT (L/min) is the equilibrium binding constant.

By plotting q_e_ as a function of ln(C_e_), we obtain a slope line B and ordinate at the origin BlnK_T_.

The theoretical parameters of the adsorption isotherms for the three metal cations with their correlation coefficients are listed in Table 4. We can note that the Freundlich and Temkin models are not suitable for modeling the adsorption of the three metal cations on the studied adsorbent. On the other hand, the Langmuir model applies well to the experimental results obtained over the entire concentration range studied. It seems that the Langmuir model is the most representative of the adsorption mechanism with correlation coefficients close to unity (R^2^ = 0.9981, 0.9988, and 0.9997 respectively for Cd(II), Cu(II), and Pb(II)). Overall, it seems that the adsorption of metal cations is done by monolayer on identical energy sites. These results are in good agreement with those in the literature, since numerous studies conducted on the adsorption of metal cations on different types of clay supports have led to the same conclusions [11,15,25].

**Table 4:** Isotherms parameters for Cd(II,) Cu(II), and Pb(II).

	Langmuir				Freundlich			Temkin			
	qe (exp)	qm,cal	KL	R2	KF	1/n	R2	KT	bT (J/mol)	B	R2
Cd(II)	5.12	5.25	0.1741	0.9981	1.4797	0.2758	0.9501	0.2006	1817.1277	1.3635	0.9850
Cu(II)	13.16	13.41	0.2344	0.9988	6.2333	0.1589	0.9761	0.0015	3370.4644	0.7351	0.9908
Pb(II)	15.70	15.90	1.0329	0.9997	9.8108	0.1105	0.8475	0.0001	3892.1190	0.6366	0.8610

The separation factors of different concentrations are collected in Table 5. All values are less than unity, implying that the Langmuir isotherm best describes the adsorption of the three metal cations on AM clay [49].

**Table 5:** TSeparation factor RL of the Langmuir isotherm.

Concentration (mg/L)	10	20	40	60	80	100	150	200
Cd(II)	0.3825	-	0.1252	0.0876	0.0680	0.0550	0.0361	0.028082
Cu(II)	0.2856	-	0.0954	0.0659	0.0504	0.0408	0.0275	0.020845
Pb(II)	-	0.0481	0.0235	0.0159	0.0122	0.0096	0.0064	0.004854

### 3.5. Thermodynamic parameters

The adsorption phenomenon is always accompanied by a thermal process which can be either exothermic (DH < 0) or endothermic (DH > 0). The value of DH is the main criterion for differentiating chemisorption from physisorption [50]. The equilibrium constant of adsorption Kd is related to the free enthalpy of the reaction DG and thus to the enthalpy DH and the entropy DS of adsorption by the relation Eq. (10):

(10)ΔG=ΔH-TΔS=-RTlnKd

It comes Eq. (11): 

(11)lnkd=ΔSR-ΔHRT

where K_d_ is the equilibrium constant, DG is the free enthalpy (J/mol), DH is the enthalpy (J/mol), DS is the entropy (J/mol K), T is the absolute temperature (K), and R is the universal gas constant (8.314 J/mol K).

**Figure 9:**
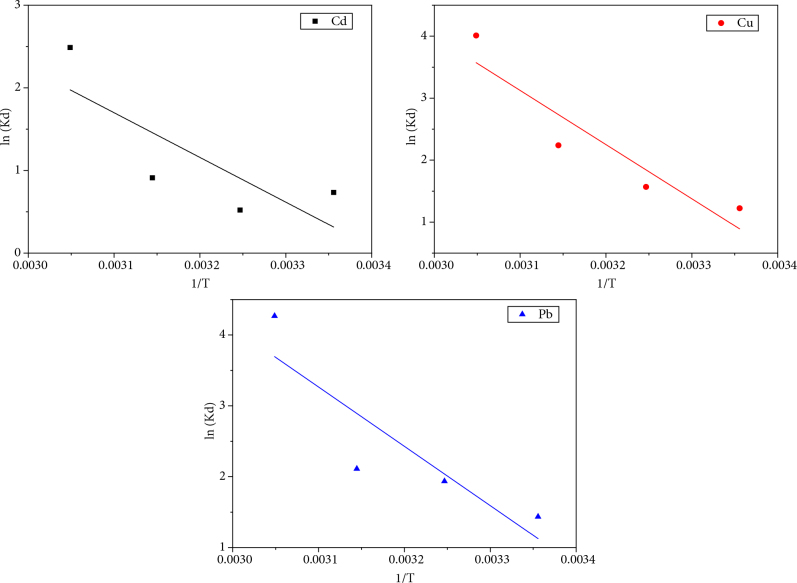
Representation of ln(K_d_) as a function of (1/T) for the three metals: (a) cadmium, (b) copper, and (c) lead.

The plots of ln(K_d_) as a function of 1/T for the metal cations Cd^2+^, Cu^2+^, and Pb^2+^ are shown respectively in Figures 9a, 9b, and 9c. The representation of ln(Kd) as a function of 1/T is a line whose slope and ordinate at the origin allow to calculate respectively the standard variations of enthalpy DH and entropy DS and free energy DG. The obtained results are presented in Table 6. 

**Table 6:** Thermodynamic parameters.

	T (K)	Kd (g/L)	∆G° (KJ mol –1)	∆H° (KJ mol–1)	∆S° (KJ mol–1 K –1)
Cd(II)	298	2.08	–1.82	44.97	0.15
	308	1.68	–1.33		
	318	2.49	–2.41		
	328	12.03	–6.78		
Cu(II)	298	3.39	–3.03	72.65	0.25
	308	4.79	–4.01		
	318	9.39	–5.92		
	328	55.15	–10.94		
Pb(II)	298	4.19	–3.55	69.61	0.24
	308	6.93	–4.96		
	318	8.24	–5.58		
	328	71.33	–11.64		

The negative values of ∆G indicate the spontaneous nature of the adsorption process. The positive values of **∆**H demonstrate the endothermic character of the adsorption process, and as they are higher than 40 kJ/mol, it is therefore a chemisorption [50]. The positive values of ∆S indicate the increase of disorder at the solid-liquid interface.

### 3.6. Adsorption mechanism

**Figure 10:**
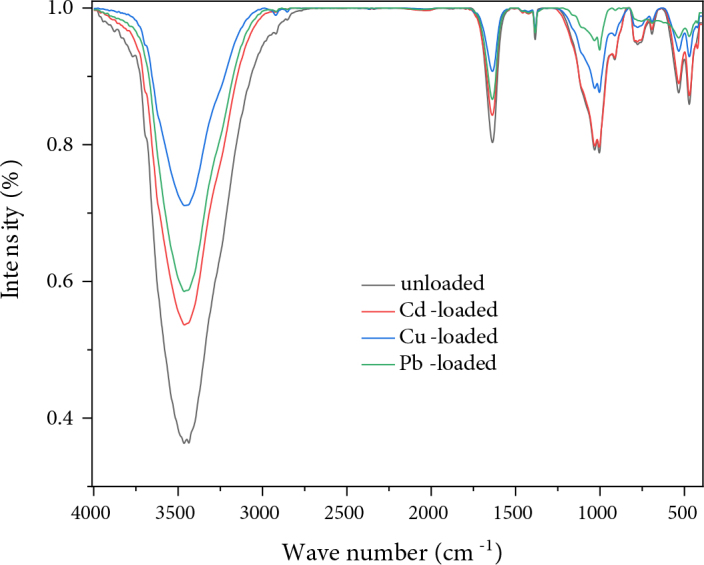
FTIR spectrum of AM clay before and after adsorption.

To elucidate the nature of the possible interaction between adsorbent/adsorbate and to identify the different functional groups involved in this interaction, FTIR spectrophotometric analyses of the unloaded and the loaded clay with Cd^2+^, Cu^2+^ or Pb^2+^ ions were carried out. The FTIR spectra are illustrated in Figure 10. It can clearly be seen that the reduction in peak size at 3436 and 1637 cm^-1^ indicates the involvement of the hydroxyl group in the adsorbent-adsorbate interaction. Thus, the reduction of peaks, which are attributed to the Si-O and Al-Al-OH group, indicates the involvement of the silanol and aluminol groups in the adsorption mechanism [51].

Possible mechanism [52]: 

2XO-+M2+→(XO)2M

with X = Si or Al.

## 4. Conclusion

The use of a Moroccan clay (AM) in removal of Cd^2+^, Cu^2+^, and Pb^2+^ ions from synthetic aqueous solutions has been studied. One of the advantages of this study was to use unmodified clay, which reduces the costs of the adsorption procedure. The following conclusions can be made from this study:

· Adsorption of Cd^2+^, Cu^2+^, and Pb^2+^ ions onto AM clay was found to be influenced by AM clay dose, contact time, initial aqueous solution pH, temperature, and initial concentration of metal ions.

· Experiments have shown that the adsorption kinetics of heavy metals onto clay follows the same order, the pseudo-second-order. 

· The adsorption isotherms of the three metals by AM clay are described satisfactorily by the Langmuir model. The maximum adsorption capacities for metal ions, using the Langmuir isotherm model equation, are 5.25, 13.41, and 15.90 mg/g, respectively, for Cd^2+^, Cu^2+^, and Pb^2+^ ions.

· Thermodynamic parameters reveal that the adsorption process of metal ions onto AM clay was spontaneous and endothermic.

Based on these results, it can be concluded that natural clay (AM) can be used as an inexpensive and effective adsorbent in removal of Cd^2+^, Cu^2+^, and Pb^2+^ ions from aqueous solutions.
